# A new data management system for the French National Registry of human alveolar echinococcosis cases[Fn FN1]

**DOI:** 10.1051/parasite/2014075

**Published:** 2014-12-22

**Authors:** Amandine Charbonnier, Jenny Knapp, Florent Demonmerot, Solange Bresson-Hadni, Francis Raoul, Frédéric Grenouillet, Laurence Millon, Dominique Angèle Vuitton, Sylvie Damy

**Affiliations:** 1 Laboratoire Chrono-Environnement, UMR/CNRS 6249 University of Franche-Comté 25000 Besançon France; 2 OSU THETA Franche-Comté Bourgogne 25000 Besançon France; 3 National Reference Centre of Alveolar Echinococcosis – FrancEchino Network – WHO Collaborating Centre for Prevention and Treatment of Human Alveolar Echinococcosis, University Hospital Centre of Besançon 25000 Besançon France

**Keywords:** Alveolar echinococcosis, Registry, Database, Interoperability of data

## Abstract

Alveolar echinococcosis (AE) is an endemic zoonosis in France due to the cestode *Echinococcus multilocularis*. The French National Reference Centre for Alveolar Echinococcosis (CNR-EA), connected to the FrancEchino network, is responsible for recording all AE cases diagnosed in France. Administrative, epidemiological and medical information on the French AE cases may currently be considered exhaustive only on the diagnosis time. To constitute a reference data set, an information system (IS) was developed thanks to a relational database management system (MySQL language). The current data set will evolve towards a dynamic surveillance system, including follow-up data (e.g. imaging, serology) and will be connected to environmental and parasitological data relative to *E. multilocularis* to better understand the pathogen transmission pathway. A particularly important goal is the possible interoperability of the IS with similar European and other databases abroad; this new IS could play a supporting role in the creation of new AE registries.

## Abstract (fr)

L’échinococcose alvéolaire (EA) est une zoonose endémique en France, due au cestode *Echinococcus multilocularis*. Le Centre National de Référence français de l’échinococcose alvéolaire (CNR-EA), lié au réseau FrancEchino est en charge de l’enregistrement de tous les cas d’EA diagnostiqués en France. Les informations administratives, épidémiologiques et médicales sur les cas d’EA français ne sont actuellement exhaustives que pour la période du diagnostic. Dans le but de constituer un jeu de données de référence, un système d’information (SI) a été développé grâce à un système de gestion de base de données relationnelle (langage MySQL). L’actuel jeu de données évoluera vers un système de surveillance dynamique, prenant en compte les données de suivi (par exemple, données d’imagerie, sérologie) et sera connecté à des données environnementales et parasitologiques, relatives à *E. multilocularis*, pour mieux comprendre les voies de transmission du pathogène. Un point particulièrement important ici est la possibilité du SI d’être interopérable avec d’autres bases de données similaires, en Europe et ailleurs ; ce nouveau SI pourrait donc servir de support à la création de nouveaux registres de l’EA.

## Introduction

Alveolar echinococcosis (AE) is a rare but severe endemic zoonosis due to the cestode *Echinococcus multilocularis*, commonly named the fox tapeworm. The main actors of the sylvatic cycle are carnivores (e.g. fox and dogs) as definitive hosts, which harbour the adult stage in their small intestine, and voles and lagomorphs as intermediate hosts, which harbour the larval stage mainly in the liver [19]. Humans are infected by eating raw vegetables or by contact with soil or carnivore fur contaminated by the parasite eggs [3]. Domestic carnivores can be involved in the parasite cycle and their close contact with humans increases the risk of parasite transmission [17]. The diagnosis of AE can only be performed several years after the contact with the parasite due to a long asymptomatic period [1]. The occurrence of the disease in humans is considered a multifactorial event. Different environmental and behavioural factors are suspected to be responsible for the infection, such as activity in agriculture, hunting or close contact with carnivores [9]. The marked increase in the fox population in Europe and the presence of foxes in the cities have increased the probability of contact with the parasite within the last three decades, as demonstrated in Switzerland [16]. Along with such external risk factors, new host-linked risks are emerging such as the high incidence rate of AE in immunosuppressed patients, suggesting an opportunistic status for the parasite [4]. The delay between first contact and diagnosis and the multifactorial causes of disease occurrence emphasize the complexity of diagnosis and the possibility of epidemiological changes over time. In most regions, disease incidence is low, and changes may either remain unnoticed or be misinterpreted at a single centre level by hospitals out of the endemic region. This would justify permanent population-based surveillance of the disease at a continental level. However, after a first attempt at establishing an observatory of cases at the European level, the EurEchinoReg project [9], and despite the inclusion of echinococcosis among those zoonoses that should be reported to the European CDC (Centre For Disease Control) and EFSA (European Food Safety Authority), AE surveillance is currently performed in each state and is rather heterogeneous in its organization and reliability [7].

In France the historical endemic area is represented, for human cases, by the eastern regions and the mountains of Massif Central; 22 French administrative divisions named *départements* at risk (DAR) of human infection by *E. multilocularis* have been defined [12]. The annual incidence is 0.026 new cases per 100,000 inhabitants (at the country level), and in 2003, French cases represented 42% of the total number of collected cases in Europe [9, 14]. Since 1997, the WHO-Collaborating Centre for Prevention and Treatment of Human Echinococcosis, located in Besançon, France, at the Regional and University Hospital Centre (CHRUB), has recorded the human cases of AE diagnosed on the French territory retrospectively for 1982–1996, then prospectively afterwards [9]. Since 2004, using different data management software types, data collection was performed within the framework of an agreement between the French Institute for Public Health Surveillance (*Institut de Veille Sanitaire*, InVS) and the FrancEchino network, and in 2012 it became part of the missions given by the InVS to the National Reference Centre for Alveolar Echinococcosis (*Centre National de Référence de l’Échinococcose Alvéolaire*, CNR-EA). This comprehensive recording of AE cases has allowed the FrancEchino registry to serve as a reliable basis for epidemiological and clinical studies [4, 9, 11, 12]. Although there is no legal notification of AE cases in France, the aim of the FrancEchino registry is to record all AE cases diagnosed in France on a population-basis and in a most exhaustive manner. This is achieved through active investigation thanks to collaborations between the CHRUB and hospital centres from the eastern, northern and central parts of France (FrancEchino Network), and by yearly requests to hospital pharmacies in charge of supplying Eskazole^®^ (albendazole), the only drug available to treat the disease, as well as to Pathology and Parasitology laboratories. Communication between the FrancEchino network partners and non-specialists in AE among health professionals all over France improves the awareness of all actors about AE and its presentation in patients. In addition to its surveillance role, the AE monitoring system may thus participate in decreasing the time to AE diagnosis in non-endemic regions and in improving the care management of patients. To achieve its objectives, the FrancEchino registry must adopt strict rules and be structured around a universal system taking into account the complex clinical and environmental aspects of AE, by integrating new data (such as co-morbidities) and must be able to connect to environmental data relevant for transmission (e.g. prevalence of *E. multilocularis* in foxes, landscape features). This has led us to move from the existing simple data file systems to a new efficient, secure and easy-to-use database which would allow the quality control of recorded data, the standardization of patient enrolment and follow-up, the integration of ecological and environmental data, and the targeted consultation of the data based on different types of users. This paper aims to describe the design of this new IS, to highlight the methodological issues, and to discuss its possible extension to other countries for integrated surveillance of AE in endemic regions of the world.

## Materials and methods

As the design of the AE registry information system (IS) is geared towards disease surveillance and clinical monitoring, as well as data use in scientific research, such a system should not be fixed and must be able to evolve over time. For that reason, we used a process called “separation of concerns (SoC)” [6] for the development of the IS. This approach separates what is to be done from how it is to be done. Through proper separation of concerns, complexity becomes manageable.

The first step was to describe all AE key data collected for the registry as patient data, medical data, epidemiological data, etc., using a conceptual model. This step required close collaboration between data managers and people in charge of modelling the current key data. The database model (i.e. the relational framework) was completed within 4 months. As it was at the beginning of our work, the documented data sets included three parts: administrative (identity, age of the patient at the time of diagnosis, sex, etc.), epidemiological (geographical location, risk factors such as trips to endemic regions, activity in agriculture, hunting, owning dogs and cats, etc.), and medical information (diagnosis date, medical and surgical history, location of the parasite lesions, surgery and drug treatment, occurrence and location of metastases, etc.).

Meanwhile, specific research projects had been implemented, which required both a common background of epidemiological and clinical data already accessible from the registry database and dedicated “modules” that could be annexed to the general background. In addition, possible extension to other research teams had to be taken into account. This first step also required clear definition of the roles of each user who could have access to the IS.

The second step was the development of the IS itself, taking the needs of users into account. The IS integrates a data management system and a web application. Its use in the area of scientific research has forced the application to be developed in a scalable manner, using technologies maintained over the long term. Thus, the relational database management system (RDBMS) MySQL was used to implement the database, an open-source RDBMS widely used in the world. The web application was developed in PHP and JavaScript.

To extract master data, that represent the entities which were agreed upon and are shared across the scientific community of AE case management, we used processes of Master Data Management (MDM) [13]. When merging the previous master data and those required in the new IS, we removed duplicates, standardized data, and incorporated rules to eliminate incorrect data from entering the system in order to create a reliable source of master data.

## Results

The application of the MDM has permitted us to introduce reference data in the IS. They are used to define a set of permissible values for fields, the majority of them being defined by standards from organizations at the national level, such as country codes as defined in ISO 3166-1 or French *département* codes as defined in the COG (for “*Code Officiel Géographique*”), the geographical official code of the INSEE (for “*Institut National de la Statistique et des Études Économiques*”), the French national institute of statistics and economic studies. The usage of standard reference data is a way to improve database interoperability. Some reference data were recovered from other databases, like organs which are the same as in tumour registries, such as the Tumour Registry of the Doubs *département*, another registry linked to the InVS and operated in the same hospital (CHRUB).

**Figure 1.**
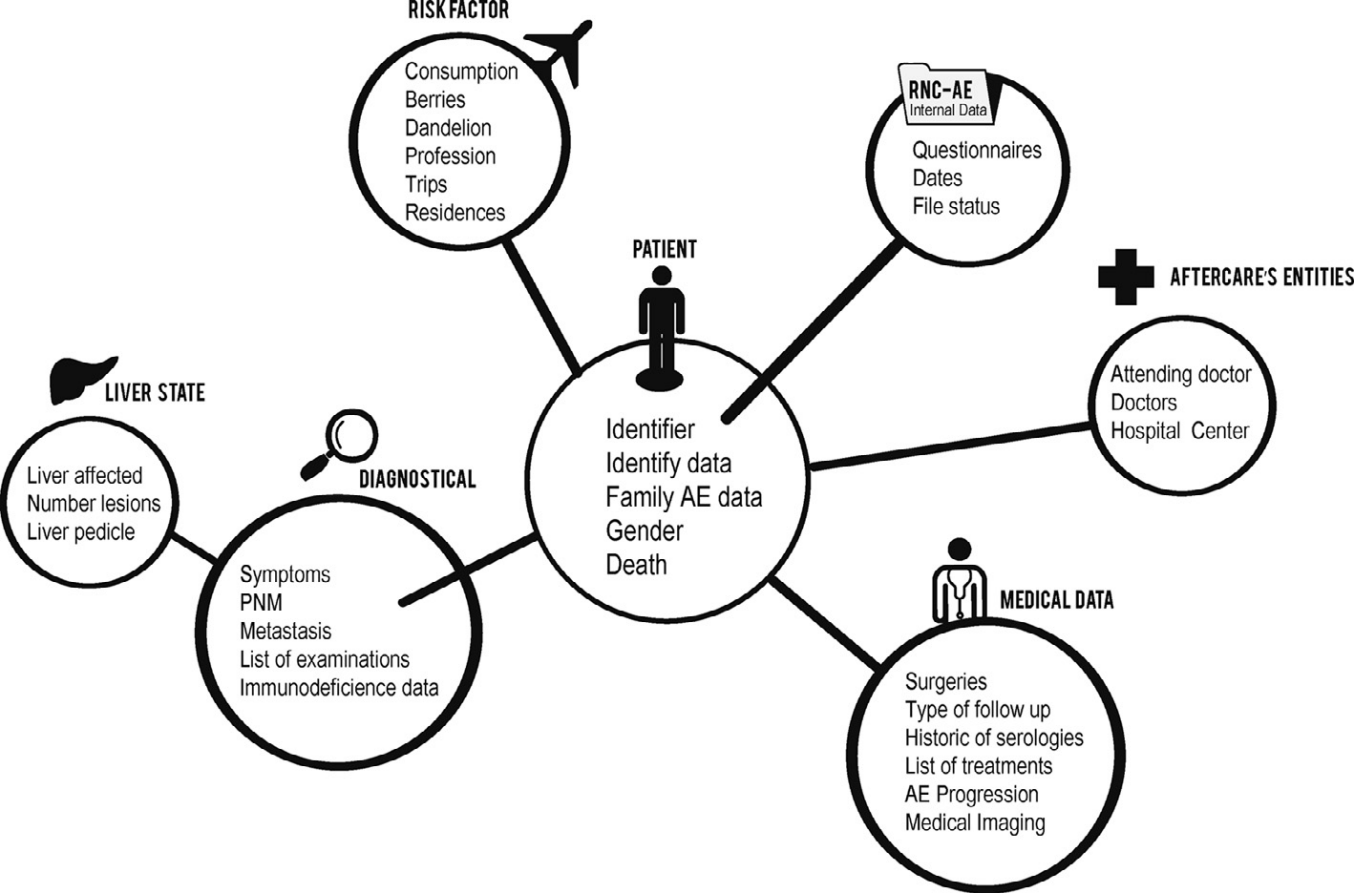
The main data parts of the information system for the FrancEchino human cases registry.

A data conceptual model was built with MERISE (an analysis and modelling methodology for information system development [2]). The model is composed of groups of main data parts (Fig. 1), e.g. patient (administrative data), epidemiological data and medical data. It provides new features like history of AE course in patients, associated diseases, care management and follow-up in terms of serology and imaging that are crucial both for diagnosis, classification and follow-up, and treatments (including surgery, medical treatment and side effects).

A database scheme was designed, allowing us to take the integration of new data types into account. If shared in the future by several research teams, depending on the level of precision case retrieval may reach, the system will allow users to define the common vs. specific items, and to analyse data in common or separately.

**Figure 2.**
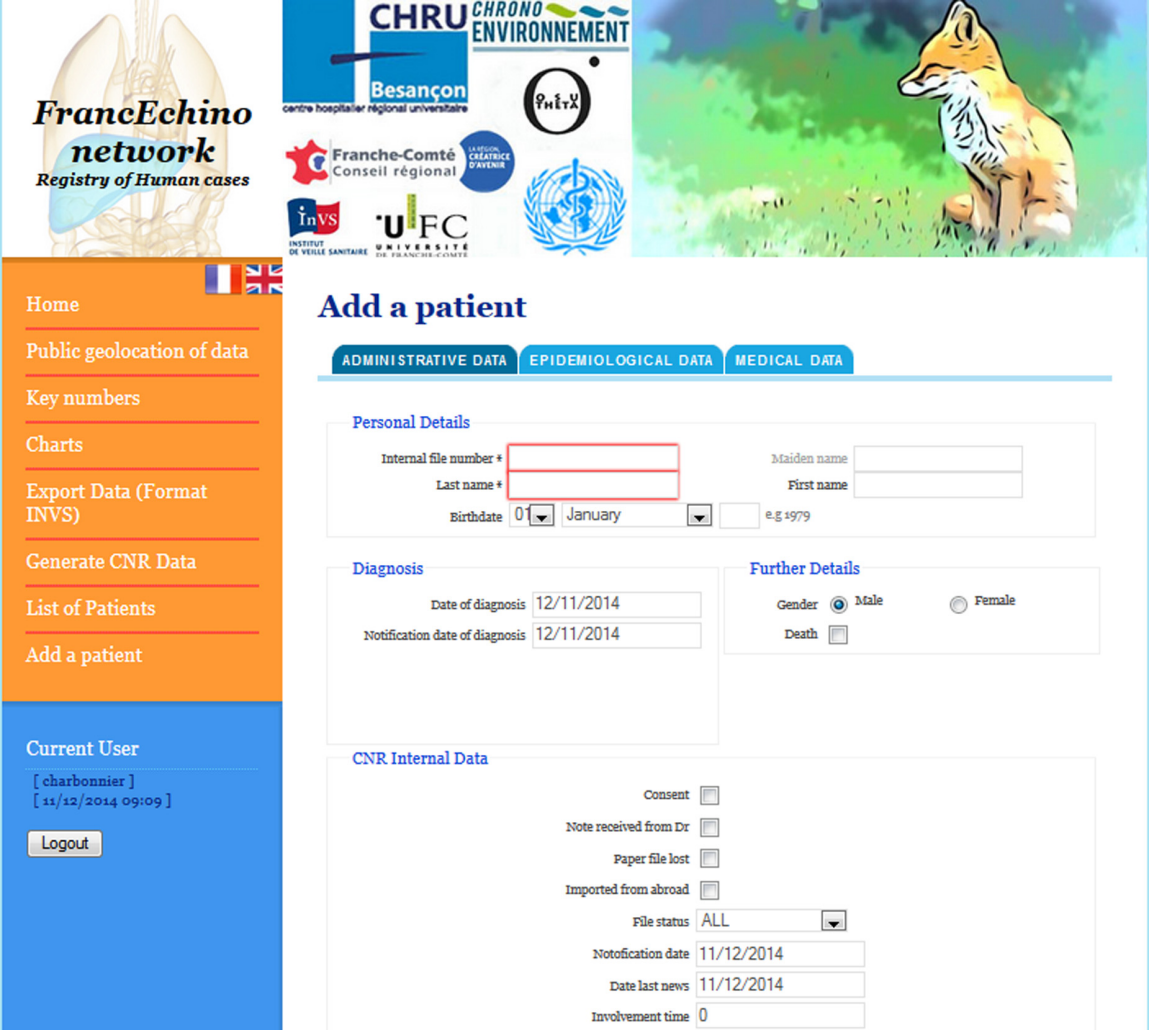
User-friendly interface: screen capture of the form accessible to the user (health professional in charge of patient) to add a patient’s file.

To guarantee accuracy (i.e. exhaustivity and absence of double recording), the registry contains patients’ names and personal data, and there are strict requirements by French national laws to secure their access (http://www.legifrance.gouv.fr/affichTexte.do;jsessionid=E0BCC13BFE62F1FB7C2C0D4213C4713F.tpdjo15v_3?cidTexte=LEGITEXT000006052581&dateTexte=20140721; http://www.recherche-biomedicale.sante.gouv.fr/files/loi/LSP-consolidee.pdf). Compliance with the recommendations of the two official French ethics committees for personal data protection has to be ensured to obtain the required authorizations to maintain a disease registry using people’s full names and addresses. These are the CNIL (for “*Commission Nationale de l’Informatique et des Libertés*”), the French data protection authority, and the CPP (for “*Comité de Protection des Personnes*”), the regional committee for patients’ legal protection. The IS thus includes many levels of security: on the web server, on the database and on the web application with a user management system. The user management system is based on roles formerly defined to use the registry but it is flexible because the architecture is based on Role-Based Access Control (RBAC) [15]. This method permitted us to define all required roles and permissions to manage user privileges. The current user management system offers three types of access: public access, FrancEchino network (or any similar type of professional network) physician access and data manager access. (1) “Public access” provides statistics, charts and maps for users. These charts and maps are automatically generated from data. (2) “FrancEchino network physician access” is available with an authentication by login and password and it offers anonymous lists of patients. (3) “Data manager access” is available with an authentication by login and password. It allows data managers to enter new data in the database after proper validation for accuracy, and to access all data. Data input by a data manager is validated beforehand by a specialist in AE, according to the procedures agreed upon between the CNR-EA and the InVS. When a data manager includes a new patient, the IS provides a form with inputs (Fig. 2). The data manager has to fill in or select reference data from a list. The records of a form are only possible if all mandatory data are filled in and if rules are checked. This new way of including a patient in the registry is easier, with a user-friendly GUI (Graphical User Interface), and it simplifies the process of data homogenization. Overall, this step improves the process of data validation. Since the anonymous data are available by authorized persons, complete non-anonymous data are exclusively localized on a local server, specifically protected from any intrusion according to the CNIL’s recommendations.

## Discussion

Zoonoses are complex diseases which combine animal and human hosts, as well as key environmental or anthropic factors which are continuously changing with time [5, 10]. An integrated system of data collection and recording must take all these aspects into account. The data management system that has been developed to improve data collection by the FrancEchino registry, in addition to complying with French National regulations, which are particularly strict, satisfies this scientific requirement and could serve as a basis for the establishment of a European or multinational registry of AE.

Applying the principle of separation of concerns to IS design results in a number of benefits such as the increase in maintainability and extensibility that can have a major impact on the adoption rate of the system. The division into components that focus on a single purpose, leads to the definition and use of components that are more easily reused in other systems, or different contexts within the same system. This principle helps users in the management of complexity by eliminating unnecessary duplication and proper responsibility allocation in entities.

The current IS provides many possibilities to export data: export to the InVS using the InVS format in spreadsheets, multicriteria export in standard formats such as “csv” format. It also enables export of charts for completing annual reports both at the National and European levels of AE surveillance. On the web application, guidelines on epidemiological and medical data registration will be provided to facilitate the assessment and recording of new cases and addition of data by data managers and FrancEchino physicians, with online access to registry codes. The IS will be online by early 2015. Along with the functionalities dedicated to clinical management and epidemiological surveillance in humans, the possibility of a specific development of this system towards the inclusion of specific research-orientated data is under study.

AE is a rare disease, and for each endemic country, the quantity of data is not a limiting factor for the choice of the system in use. The number of French human cases recorded in the FrancEchino registry with a diagnosis of AE between January 1982 and December 2013 was 575 (CNR-EA data). The highest incidence rates in France are currently recorded in five *départements* (Doubs, Haute-Saône, Jura, Vosges and Haute-Savoie) with an annual incidence higher than two new cases per 100,000 inhabitants, which represents 60% of cases recorded in France. Treatment and monitoring of the disease in humans, with long-term follow-up, represent a significant economic burden for countries [18], because of the long-term treatment and costly procedures such as liver transplantation. Long-term follow-up of each patient is therefore a factor for the incremental increase of data with time. Patient management and treatment need to be harmonized between clinical centres in Europe, as was emphasized recently [8]. A key objective was thus to develop an AE registry with standardized protocols, master and reference data to allow harmonized data management and research on AE epidemiology first at a European then at world level. The IS we developed well fits with such a requirement. At the time of writing this article, sharing this IS to include AE data from Germany is scheduled. As Germany is a traditionally endemic area with the second largest set of data regarding AE and new emerging characteristics (spreading of fox and human infection over the country, possible transmission of the parasite from foxes to humans in cities…), this common IS could become the core system for AE surveillance in Europe. Adoption of the same database system to record cases in the most endemic areas of China is also planned. A meeting of all interested groups involved in AE surveillance is scheduled for April 2015; at this meeting, final decisions on points still debated, for instance on shared key definitions (date of diagnosis, imaging classification in addition to PNM classification – parasite lesion, neighbouring organ invasion, metastases) and procedures for case-recording and cross-confirmation of cases to reach completeness, adapted to each country, should be agreed upon.
